# Tranexamic acid to prevent anastomotic leak after rectal cancer surgery: protocol for a feasibility trial with embedded mechanistic analysis of the microbiome

**DOI:** 10.1136/bmjopen-2025-114040

**Published:** 2026-09-11

**Authors:** Jack A Helliwell, Caroline H Chilton, Janine Bestall, Andrew Kirby, Philip Quirke, Henry M Wood, Deborah D Stocken, David Jayne

**Affiliations:** 1 University of Leeds Leeds Institute of Medical Research, Leeds, UK; 2 University of Leeds Leeds Institute of Health Sciences, Leeds, UK; 3 University of Leeds Leeds Institute of Clinical Trials Research, Leeds, UK

**Keywords:** Colorectal surgery, Gastrointestinal Microbiome, Clinical trials

## Abstract

**Introduction:**

Anastomotic leak is a major complication after anterior resection for rectal cancer, with reported rates of 10–15%. Recent evidence implicates the gut microbiome, where specific bacteria colonise the anastomotic site and secrete collagenase that weakens healing tissue. Preclinical studies demonstrate that tranexamic acid can inhibit this process when delivered rectally into the bowel lumen. This protocol outlines a feasibility trial of rectally administered tranexamic acid delivered via rectal catheter to the anastomotic site after anterior resection, accompanied by embedded microbiome and qualitative substudies.

**Methods and analysis:**

This is an open-label, single-centre, randomised controlled feasibility trial. 45 patients undergoing anterior resection for rectal or sigmoid cancer will be randomised 2:1 to receive tranexamic acid or sterile water, administered intraoperatively and on postoperative days 1–3 via a rectal catheter. Feasibility outcomes include assessment of recruitment, intervention adherence, protocol compliance and safety. Exploratory clinical outcomes include anastomotic leak and other postoperative complications. A mandatory microbiome substudy will characterise changes in microbial composition and bacterial collagenase activity, providing mechanistic insight into treatment response. An optional qualitative substudy will explore acceptability of the intervention from the perspectives of patients and healthcare professionals.

**Ethics and dissemination:**

The protocol was approved by the North West Liverpool Central Research Ethics Committee and the Medicines and Healthcare products Regulatory Agency on 30 October 2024 (24/NW/0254). Recruitment commenced on 7 January 2025 following receipt of site-specific approvals. Feasibility, mechanistic and qualitative findings will be disseminated through peer-reviewed publications, academic conferences and stakeholder engagement activities. The results will inform progression to a definitive phase III trial and shape key elements of study design.

**Trial registration number:**

ISRCTN13727659.

**STRENGTHS AND LIMITATIONS OF THIS STUDY**

- Evaluates a novel, biologically plausible intervention with strong preclinical rationale, repurposing a widely used drug that has an established safety profile.
- Directly addresses key uncertainties of feasibility, safety and acceptability of rectal tranexamic acid delivered via rectal catheter.
- Incorporates embedded microbiome and qualitative substudies, providing both mechanistic insight and patient and clinician perspectives to inform future trial design.
- Given the limited sample size, the study is neither intended nor sufficiently powered to detect differences in anastomotic leak rates.

## Introduction

 In the UK, approximately 30 000 patients undergo surgery for colorectal cancer every year.^1^ Surgical management typically involves resection of the diseased bowel segment and restoration of gastrointestinal continuity by anastomosis. While potentially curative, this approach carries the risk of serious complications, the most significant being anastomotic leak. Anastomotic leak occurs when the anastomosis fails to heal adequately, resulting in leakage of intestinal contents into the abdominal cavity.^2^

The consequences of anastomotic leak can be severe. Patients may develop intra-abdominal abscesses, faecal peritonitis, multiorgan failure or death. Even when not fatal, leaks are associated with prolonged hospitalisation, increased morbidity and significant healthcare costs.^3–5^ Despite adherence to technical principles such as constructing a tension-free, well-perfused anastomosis, leak rates remain high. Following anterior resection, leak rates of 10–15% are reported, which are higher than for right sided resections.^6 7^

Increasing evidence suggests the gut microbiome may play a causal role in anastomotic leak. Certain commensal organisms, including *Enterococcus faecalis* and *Pseudomonas aeruginosa*, can colonise the anastomotic site and secrete collagen-degrading enzymes that weaken the structural integrity of healing tissue.^8^ Targeted modulation of the microbiome may therefore represent a novel strategy to prevent anastomotic leak.^9^

Tranexamic acid is a widely used antifibrinolytic agent that has been shown in preclinical studies to reduce bacterial collagenase production. Collagenase-producing bacteria exploit the host fibrinolytic system by activating plasminogen present within local anastomotic tissues, which upregulates collagenase activity and accelerates collagen breakdown.^10^ Tranexamic acid inhibits this pathway and reduces bacterial mediated collagen degradation in vitro and anastomotic leak in animal models.^11^

To achieve these rectal microbiome effects, local delivery of tranexamic acid into the bowel lumen is required. In patients undergoing anterior resection, where the anastomosis is in the rectum, this can be achieved using a rectal catheter. However, rectal administration of tranexamic acid has not yet been evaluated in humans. Before a definitive randomised controlled trial can be undertaken, it is necessary to establish whether this intervention is safe, feasible and acceptable, and to determine whether recruitment, intervention and follow-up processes for such a trial can be successfully delivered.

## Aims and objectives

The aim of this study is to assess the feasibility, acceptability and safety of rectally administered tranexamic acid and to determine whether a definitive randomised controlled trial can be undertaken to investigate the role of tranexamic acid in preventing anastomotic leak.

Objectives of the study include:

- To identify participant, healthcare worker and logistical barriers to recruitment.
- To estimate realistic site-level recruitment rates.
- To assess participant and healthcare worker compliance to the intervention and trial processes.
- To obtain preliminary safety and efficacy data on rectally administered tranexamic acid.
- To evaluate the feasibility of conducting a phase III randomised controlled trial.

## Methods

### Study design

**Figure 1:**
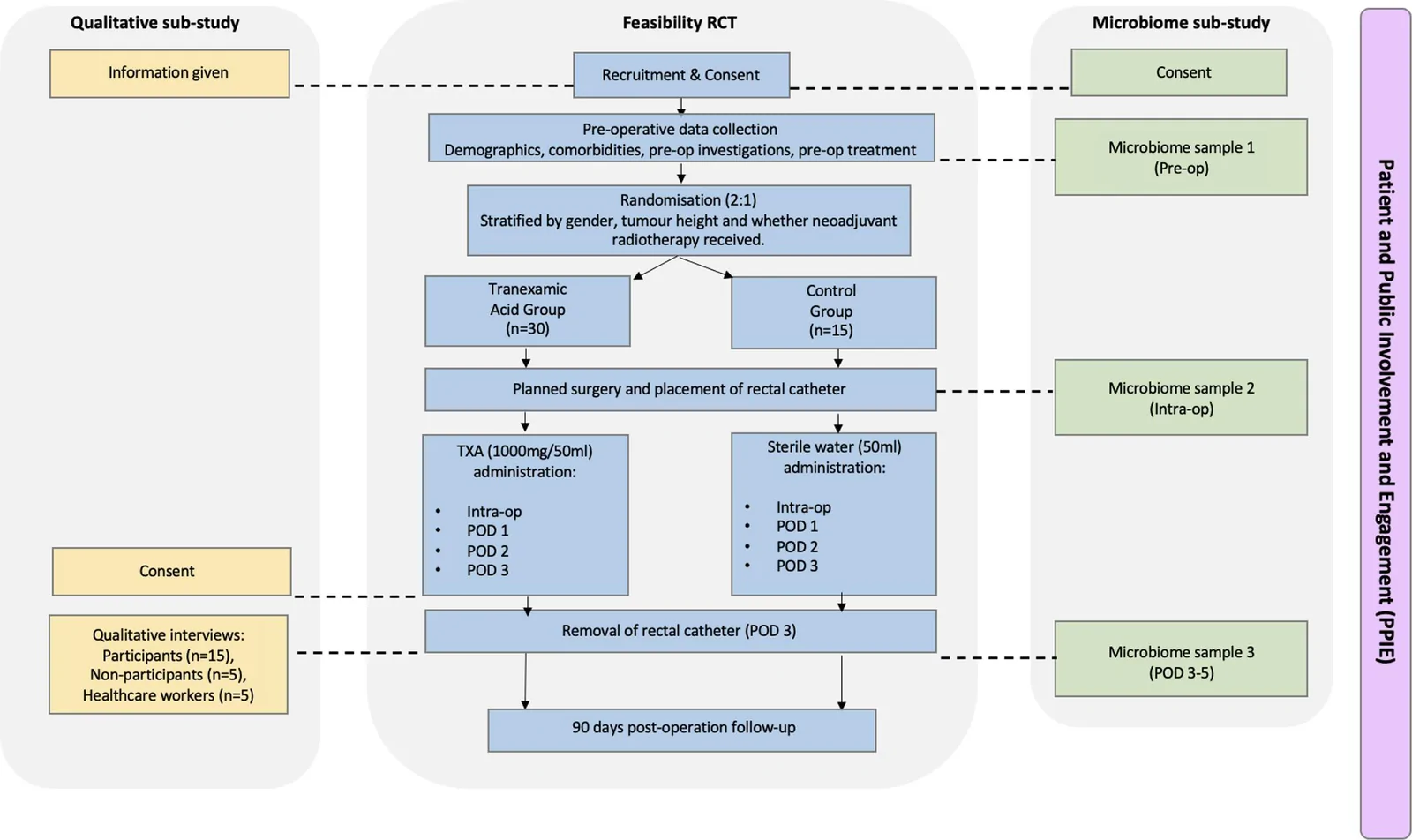
Study flow chart. Flow of participants through the feasibility trial. The diagram shows key stages including recruitment, randomisation, delivery of allocated intervention, follow-up, and involvement in embedded microbiome and qualitative sub-studies. TXA, tranexamic acid; POD, post-operative day; RCT, randomised controlled trial; TXA, tranexamic acid.

This study is a single-centre, parallel-group, randomised controlled feasibility trial evaluating rectally administered tranexamic acid to prevent anastomotic leak following anterior resection for rectal or sigmoid cancer (figure 1). 45 participants will be randomised in a 2:1 ratio to receive either tranexamic acid (1000 mg/50 mL) or sterile water (50 mL), administered intraoperatively and on postoperative days 1–3 via a rectal catheter positioned at the anastomosis. All participants will be followed up for 90 days after surgery.

The trial incorporates two embedded substudies: (1) a mandatory microbiome substudy to provide mechanistic insight into treatment response, and (2) an optional qualitative substudy to explore acceptability of the intervention and trial processes.

### Trial setting

The trial will be conducted at St James’s University Hospital, Leeds Teaching Hospitals NHS Trust, UK. This tertiary referral centre performs approximately 80–100 anterior resections annually and has established infrastructure for colorectal surgical research. Recruitment commenced on 7 January 2025, with study completion anticipated on 1 January 2027

### Trial participants

To be considered eligible for the study, patients must satisfy all of the following inclusion criteria:

- Aged ≥18 years.
- Able to provide written informed consent.
- Biopsy proven adenocarcinoma of the rectum or sigmoid colon, or high-grade dysplasia with endoscopic or radiological features suggestive of cancer.
- Planned curative resection by high or low anterior resection.
- Suitable for elective open, laparoscopic or robotic surgery.
- American Society of Anesthesiologists grade ≤3.
- Able and willing to comply with trial procedures, including contraception requirements where applicable.

Presence of any of the following exclusion criteria will preclude participation in the trial:

- Planned resection without anastomosis (eg, Hartmann’s procedure, abdominoperineal resection).
- Coexistent colorectal pathology (eg, inflammatory bowel disease).
- Contraindication to tranexamic acid (eg, known allergy, previous venous or arterial thrombosis, seizure disorder, disseminated intravascular coagulation, severe renal impairment).
- Pregnant, breastfeeding or likely to become pregnant within 3 months of surgery.

### Recruitment and consent

The timing of study procedures, clinical assessments and data collection points are summarised in online supplemental table 1. Potential participants will be identified through weekly colorectal multidisciplinary team meetings and systematic screening of surgical waiting lists. Those meeting the eligibility criteria will be approached by a member of the research team and provided with both verbal and written information about the study, including the participant information sheet.

Written informed consent will be obtained using the approved consent form by trial researchers trained in Good Clinical Practice. Consent will cover participation in the trial, administration of the allocated intervention, collection of clinical data and mandatory microbiome sampling. Separate consent will be sought for the optional qualitative substudy, typically during the participant’s attendance at the follow-up clinical appointment.

### Randomisation

Randomisation and allocation will be undertaken after confirmation of eligibility and completion of written informed consent. Allocation will be managed using a secure, 24-hour web-based randomisation service provided by Sealed Envelope.

Participants will be randomised in a 2:1 ratio to the tranexamic acid group or the control group. This allocation ratio was chosen to maximise knowledge of the intervention’s feasibility, safety and acceptability within a small sample size, while still exploring potential barriers to recruitment and randomisation, and benchmarking the control group’s experience and microbiome profile.

A minimisation algorithm with a random component will be applied to ensure balance across three prognostic factors known to influence anastomotic leak risk: sex (male; female), distance of the lower edge of the tumour from the anal verge (<8 cm; ≥8 cm) and receipt of neoadjuvant therapy (yes; no).

### Blinding

This trial will be conducted as an open-label study in which participants, the clinical team and the research team will be aware of the allocated intervention. An unblinded design was selected because the primary objectives are to assess feasibility, acceptability and safety of rectally administered tranexamic acid, with a particular focus on recruitment, compliance and practical barriers to delivery. As the study is not powered to evaluate treatment efficacy and does not involve subjective outcome measures, the absence of blinding is not anticipated to impact interpretation of any of the predefined progression criteria.

### Intervention

All participants will undergo anterior resection, according to the surgeon’s standard technique using an open, laparoscopic or robotic approach. At the end of the procedure, a rectal catheter will be inserted with the tip positioned just proximal to the anastomosis by the operating surgeon, allowing direct delivery of the allocated treatment to the anastomotic site.

Participants randomised to the intervention arm will receive tranexamic acid (1000 mg/50 mL) via the catheter immediately after creation of the anastomosis and once daily on postoperative days 1–3. Participants in the control arm will receive sterile water (50 mL) at the same time points. Sterile water is chosen as an inert comparator to replicate the administration procedure without introducing pharmacological effects, enabling assessment of feasibility, safety and acceptability while providing a baseline for mechanistic microbiome analyses.

The rectal catheter will be removed following administration of the final dose on postoperative day 3. If the rectal catheter becomes dislodged before all scheduled doses have been given, treatment will be discontinued, and treatment duration recorded.

### Safety monitoring

Adverse events will be considered for all participants from the signing of informed consent until 90 days after the last dose administration of investigational medicinal product. The active monitoring period for adverse events will be from the day of surgery until 24 hours following discontinuation of the study drug. Outside this period, investigators are still required to report any adverse events they become aware of.

Adverse events will be assessed for seriousness, causality and expectedness. All serious adverse events judged to be potentially related to the investigational medicinal product will be promptly reported to the University of Leeds as Sponsor (online supplemental file).

### Study outcomes and progression criteria

The primary outcome of this feasibility trial is to determine whether prespecified progression criteria are met to support progression to a phase III randomised controlled trial evaluating the clinical and cost-effectiveness of rectal tranexamic acid to prevent anastomotic leak.

Progression criteria address the key feasibility domains of recruitment, intervention adherence, retention and safety:

- Recruitment: proportion of the target sample of 45 participants recruited.
- Intervention adherence: mean number of scheduled treatment doses received per participant.
- Retention: proportion of participants completing 90-day follow-up, including completeness of clinical outcome data and rates of withdrawal.
- Safety: adverse events, assessed for seriousness, causality and relatedness and summarised by type and incidence.

For recruitment, adherence and retention, quantitative thresholds will be applied using a stop-amend-go framework (table 1). ‘Stop’ outcomes indicate that progression to a definitive trial is not advisable unless modifiable barriers are identified and further feasibility testing is undertaken. ‘Amend’ outcomes indicate that modifications are required before proceeding to a larger study. ‘Go’ outcomes support progression to a definitive trial.

**Table 1:** Progression criteria

Domain	Outcome measure	Stop	Amend	Go
Recruitment	Proportion of target sample (45 participants) recruited within 21 months.	<75% (<34 participants)	75–99% (34–44 participants)	100% (45 participants)
Adherence	Mean number of doses received per participant.	<50% (<2 doses on average)	50–74% (2–3 doses on average)	≥75% (≥3 doses on average)
Retention	Proportion of participants completing 90-day follow-up.	<70%	70–85%	>85%

Table 1 stop-amend-go thresholds for key feasibility domains. ‘Stop’ indicates that progression to a definitive trial is not advisable unless modifiable barriers are addressed and further feasibility testing is undertaken; ‘Amend’ indicates that modifications are required before proceeding to a larger study; ‘Go’ supports progression to a definitive trial. Safety will be considered separately through independent review of cumulative adverse event data by the Trial Oversight Committee (TOC) and will inform overall progression decision.

Safety is not included within the stop-amend-go framework because progression cannot be determined using numerical thresholds alone. The implications of adverse events depend on their frequency, severity, expectedness and causality. Accordingly, cumulative safety data, including that related to medicinal product and rectal catheter, will be reviewed by the independent TOC throughout the study and at study completion to determine whether any safety concerns preclude progression to a definitive phase III trial.

The decision to progress will be based on the overall assessment across all feasibility domains, informed by both quantitative thresholds and safety review. No single feasibility domain will determine progression in isolation. Where one or more domains fall within the ‘amend’ range, progression will be informed by consideration of the underlying reasons and whether these can be addressed through modifications to the trial design or conduct. In addition, insights from the mechanistic microbiome substudy and qualitative substudy will supplement this assessment by providing complementary biological and experiential perspectives on feasibility, acceptability and the potential treatment effect.

Clinical outcome data will also be collected to explore variability and inform the design of a potential future trial, including:

- Anastomotic leak, defined and graded according to the International Study Group for Rectal Cancer criteria.^12^
- Postoperative complications assessed using the Clavien-Dindo classification.^13^

### Sample size

As a feasibility study, no formal power calculation has been performed. A total of 45 participants will be recruited, with 30 allocated to the tranexamic acid group and 15 to the control group. This sample size is sufficient to estimate feasibility outcomes, including recruitment, intervention adherence, retention and safety, to inform progression to a definitive trial.^14^

The 30 participants in the intervention group will also allow preliminary assessment of microbiome changes and associated bacterial collagenase activity, as well as collection of complication rates, providing mechanistic and clinical data to support the design of a potential future definitive trial.^14^

### Trial substudies

#### Microbiome substudy

All participants enrolled in the feasibility trial will also take part in a mandatory microbiome substudy, with consent obtained in parallel to the main trial. This substudy is designed to characterise changes in microbial composition and bacterial collagenase activity, providing mechanistic insight into treatment response.

Microbiome samples will be collected using rectal swabs from the rectal lumen at three time points: (1) preoperatively, (2) on the day of surgery and (3) on postoperative day 3–5.

Two exploratory analyses will be undertaken:

- 16S rRNA sequencing to determine how microbial populations change in response to tranexamic acid.
- Bacterial collagenase assay to quantify changes in collagenase production in response to tranexamic acid.^15^

#### Qualitative substudy

An optional qualitative substudy will be conducted to explore the acceptability of both the intervention and trial processes.

A maximum variation sampling approach will be used to ensure diversity of views by age, sex and treatment arm. Up to 15 trial participants will be interviewed (10 participants from the tranexamic acid group, and 5 participants from the control group), alongside 5 individuals who declined participation in the main trial to capture reasons for non-participation. In addition, five surgeons or healthcare professionals will be interviewed to provide their experiences of the intervention for patients under their care.

Semi-structured interviews, guided by pre-designed topic guides, will be conducted by a study investigator trained in qualitative research methods in hospital or outpatient settings and will last approximately 30–45 min. Interviews will be audio-recorded, transcribed verbatim and thematically analysed to identify key factors influencing trial feasibility and acceptability.^16^

### Data collection and management

Study data will be recorded using electronic case report forms (eCRFs) completed by trained research staff. eCRFs will be stored on a password-protected online database (Sealed Envelope). Each participant will be allocated a unique study identifier, and all data will be recorded and stored under this code to protect confidentiality. Original consent forms and other paper-based study documents will be retained in a secure, restricted-access research area, accessible only to authorised study personnel.

### Statistical analysis plan

All analyses will follow a predetermined statistical analysis plan and will be overseen by a qualified medical statistician.^17^ Baseline characteristics, recruitment, feasibility data and clinical outcomes will be presented descriptively as rates (categorical) and means (continuous) with 95% CIs. Feasibility outcomes will be summarised overall and descriptively by treatment allocation, where appropriate. No formal statistical hypothesis testing across randomised groups will be conducted. All analyses will be performed for intention-to-treat and per-protocol populations. Missing data will be reported but not imputed. Feasibility data will inform the decision to progress to a definitive trial according to the predefined progression criteria. The trial will be reported according to the Consolidated Standards of Reporting Trials 2025 Checklist.^18^

### Trial management and oversight

As a clinical trial of an investigational medicinal product, the study has a structured oversight framework to ensure safe, compliant conduct and support assessment of feasibility objectives (online supplemental file).

The Trial Management Group (TMG) will oversee day-to-day trial operations, including recruitment, follow-up, adherence to study procedures, consent, data collection, safety reporting and dissemination of results. The TMG will meet at least quarterly to review trial progress.

An independent TOC will provide impartial oversight of trial conduct. The TOC will include two independent clinicians, an independent methodologist and a patient and public involvement representative, all of whom are independent of the TMG, Sponsor and funder. The TOC will review study conduct, monitor safety and ethical compliance and advise the TMG of operational and governance issues, meeting at least annually. Should significant safety concerns arise, the TOC may recommend modification, temporary suspension or early termination of the study to the Sponsor.

The study Sponsor (University of Leeds) will perform quality assurance monitoring at predefined milestones: study initiation, after approximately 30% and 60% of participants are recruited and at study close-out. Monitoring will include verification of consent forms, source data and review of serious adverse events to ensure protocol compliance.

### Ethics and governance

Ethical approval was obtained from the North West Liverpool Central Research Ethics Committee and the Medicine and Healthcare products Regulatory Agency on 30 October 2024 (24/NW/0254). The study was prospectively registered with the ISRCTN registry on 31 October 2024 prior to the start of recruitment (ISRCTN13727659; https://www.isrctn.com/ISRCTN13727659). Recruitment opened on 7 January 2025 following receipt of site-specific approvals (online supplemental file). The present manuscript is reported according to the Standard Protocol Items: Recommendations for Interventional Trials 2025 checklist (online supplemental table 2).^19^

### Patient and public involvement

A dedicated Patient Advisory Group of five individuals with lived experience of colorectal surgery has been established and will meet throughout the study. The Patient Advisory Group has directly informed key aspects of the trial, including the overall study design, patient-facing materials and the method of rectal drug delivery via rectal catheter. The group will also provide ongoing input into recruitment strategies, trial conduct and dissemination of findings, helping to ensure the study remains patient-centred and accessible.

### Dissemination

Findings from the study, including microbiome and qualitative data, will be disseminated to both professional and patient audiences. Dissemination will occur through peer-reviewed publications, presentations at academic conferences and stakeholder engagement activities, ensuring the study results are widely accessible and shared with diverse, including hard to research, research communities.

## Discussion

This protocol describes a feasibility trial of rectally administered tranexamic acid to prevent anastomotic leak after anterior resection for rectal and sigmoid cancer. The study combines translational microbiome science with surgical innovation, exploring whether a widely used, established drug can be repurposed and delivered locally to modify a microbiome-mediated pathway implicated in anastomotic leak.

Tranexamic acid has been used safely for decades in surgical practice, most commonly given intravenously or orally to prevent and treat bleeding. Reported adverse events are generally mild, and serious complications are rare. In this trial, rectal administration represents an off-label route, designed to achieve high local concentrations at the anastomotic site while limiting systemic absorption. Pharmacokinetic studies of topical and rectal administration have demonstrated very low serum concentrations after local delivery—well below the threshold required to impair systemic fibrinolysis—providing reassurance that the risk of systemic side effects is minimal.^20 21^

Delivery will be facilitated by a rectal catheter, enabling precise administration of tranexamic acid into the bowel lumen at the site of the anastomosis. Rectal catheters are widely used following anterior resection and have a reassuring safety profile, including potential reductions in anastomotic leak through lowered endoluminal pressure and temporary faecal diversion.^22^ The novel aspect of this study is the use of the catheter to deliver a therapeutic agent to the anastomosis, for which there is limited precedent. A single-centre study demonstrated that repeated administration of an antibiotic solution via rectal catheter after rectal cancer surgery was feasible and safe, with no intervention-related adverse events.^23^ These findings are encouraging, but they do not remove uncertainty: questions remain around catheter retention, tolerance and acceptability, and addressing these practical issues is a central objective of this feasibility trial.

Preclinical data provide a strong mechanistic rationale for clinical testing. In vitro work has shown that collagenase-producing organisms such as *E. faecalis* and *P. aeruginosa* degrade collagen at the anastomotic site by exploiting the host fibrinolytic system via plasminogen activation.^10^ Tranexamic acid inhibits this pathway, reducing bacterial-mediated collagenolysis. In a mouse model of colorectal anastomosis, local administration of tranexamic acid by rectal enema reduced collagenase activity and improved anastomotic healing compared with controls.^11^ Supporting the clinical relevance of this mechanism, a recent exploratory analysis of the National Institute of Health and Care Research IntAct trial, which evaluated intraoperative fluorescence angiography to prevent anastomotic leak, demonstrated that collagenase-producing organisms were detectable in up to half of patients following colorectal surgery.^24^

Before a definitive phase III trial can be undertaken, several uncertainties must be resolved. These include whether rectal administration of tranexamic acid is safe in humans undergoing anterior resection, whether a catheter-based delivery is technically feasible and acceptable, and whether both participants and healthcare professionals are willing to engage with recruitment and other study processes. The current feasibility trial is specifically designed to test these aspects, using prespecified progression criteria to determine whether a larger trial is deliverable. The embedded substudies will provide complementary insights. Microbiome analyses will explore the mechanistic basis of tranexamic acid’s effects, generating pilot data that could potentially inform biomarker-driven endpoints in future studies. The qualitative substudy will capture the perspectives of patients, non-participants and healthcare professionals, providing crucial insight into acceptability and practical barriers to implementation. Together, these components will strengthen interpretation of the feasibility outcomes.

Strengths of the study are recognised. It evaluates a novel, biologically plausible intervention with strong preclinical rationale. The trial addresses clearly defined feasibility domains and uses prespecified progression criteria to ensure objective decision-making regarding progression to a definitive trial. The inclusion of embedded microbiome and qualitative substudies provides mechanistic and experiential data that complement the feasibility outcomes and help support the case for a future multicentre trial. However, there are limitations. The study has a small sample size and is not powered to detect differences in anastomotic leak or other clinical outcomes. It is single-centre, so careful consideration will need to be given to generalisability, particularly regarding rectal catheter placement and centre-level recruitment processes. The microbiome and qualitative substudies, while informative, will provide exploratory data rather than definitive conclusions.

This trial will establish whether rectally administered tranexamic acid is safe, acceptable and a logistically viable intervention in the context of anterior resection for cancer. If prespecified progression criteria are met, it will provide a foundation for a definitive multicentre phase III trial powered to assess clinical and cost-effectiveness. By addressing key uncertainties, while recognising the inherent limits of feasibility work, this study represents an important step toward developing a novel, microbiome-targeted strategy to reduce anastomotic leak.

## Supplementary material

10.1136/bmjopen-2025-114040 online supplemental file 1

## References

[1] National Bowel Cancer Audit (NBOCA) (2025). National cancer audit collaborating centre. https://www.natcan.org.uk/audits/bowel/.

[2] Helliwell JA, Quyn A, Jayne DG (2026). Anastomotic leak after colorectal surgery: current landscape and future directions. Br J Surg.

[3] Boccola MA, Buettner PG, Rozen WM (2011). Risk factors and outcomes for anastomotic leakage in colorectal surgery: a single-institution analysis of 1576 patients. World J Surg.

[4] Vonlanthen R, Slankamenac K, Breitenstein S (2011). The impact of complications on costs of major surgical procedures: a cost analysis of 1200 patients. Ann Surg.

[5] Ma L, Pang X, Ji G (2020). The impact of anastomotic leakage on oncology after curative anterior resection for rectal cancer. Medicine (Baltimore).

[6] Park JS, Huh JW, Park YA (2016). Risk Factors of Anastomotic Leakage and Long-Term Survival After Colorectal Surgery. Medicine (Baltimore).

[7] Kang CY, Halabi WJ, Chaudhry OO (2013). Risk factors for anastomotic leakage after anterior resection for rectal cancer. JAMA Surg.

[8] Alverdy JC, Hyoju SK, Weigerinck M (2017). The gut microbiome and the mechanism of surgical infection. Br J Surg.

[9] Helliwell JA, Sciberras P, Dosis A (2026). Modulation of the gut microbiota as a novel strategy to prevent anastomotic leak after colorectal surgery: Systematic scoping review. Colorectal Dis.

[10] Jacobson RA, Wienholts K, Williamson AJ (2020). *Enterococcus faecalis* exploits the human fibrinolytic system to drive excess collagenolysis: implications in gut healing and identification of druggable targets. Am J Physiol Gastrointest Liver Physiol.

[11] Jacobson RA, Williamson AJ, Wienholts K (2021). Prevention of Anastomotic Leak Via Local Application of Tranexamic Acid to Target Bacterial-mediated Plasminogen Activation: A Practical Solution to a Complex Problem. Ann Surg.

[12] Rahbari NN, Weitz J, Hohenberger W (2010). Definition and grading of anastomotic leakage following anterior resection of the rectum: a proposal by the International Study Group of Rectal Cancer. Surgery.

[13] Dindo D, Demartines N, Clavien P-A (2004). Classification of Surgical Complications. Ann Surg.

[14] Browne RH (1995). On the use of a pilot sample for sample size determination. Stat Med.

[15] Guyton KL, Levine ZC, Lowry AC (2019). Identification of Collagenolytic Bacteria in Human Samples: Screening Methods and Clinical Implications for Resolving and Preventing Anastomotic Leaks and Wound Complications. Dis Colon Rectum.

[16] Gale NK, Heath G, Cameron E (2013). Using the framework method for the analysis of qualitative data in multi-disciplinary health research. BMC Med Res Methodol.

[17] Gamble C, Krishan A, Stocken D (2017). Guidelines for the Content of Statistical Analysis Plans in Clinical Trials. JAMA.

[18] Hopewell S, Chan A-W, Collins GS (2025). CONSORT 2025 statement: updated guideline for reporting randomised trials. BMJ.

[19] Chan A-W, Boutron I, Hopewell S (2025). SPIRIT 2025 statement: updated guideline for protocols of randomised trials. The Lancet.

[20] Ausen K, Pleym H, Liu J (2019). Serum Concentrations and Pharmacokinetics of Tranexamic Acid after Two Means of Topical Administration in Massive Weight Loss Skin-Reducing Surgery. Plast Reconstr Surg.

[21] Almer S, Andersson T, Ström M (1992). Pharmacokinetics of tranexamic acid in patients with ulcerative colitis and in healthy volunteers after the single instillation of 2 g rectally. J Clin Pharmacol.

[22] Choy KT, Yang TWW, Heriot A (2021). Does rectal tube/transanal stent placement after an anterior resection for rectal cancer reduce anastomotic leak? A systematic review and meta-analysis. Int J Colorectal Dis.

[23] Wirth U, Rogers S, Haubensak K (2018). Local antibiotic decontamination to prevent anastomotic leakage short-term outcome in rectal cancer surgery. Int J Colorectal Dis.

[24] Helliwell JA, Chilton CH, Young C (2025). Perioperative changes in the microbiome during rectal cancer surgery: exploratory analysis of the National Institute for Health and Care Research (NIHR) IntAct trial. *Br J Surg*.

