# Supplementary material for: Tranexamic acid to prevent anastomotic leak after rectal cancer surgery: protocol for a feasibility trial with embedded mechanistic analysis of the microbiome
Source: BMJ Open. 2026 Sep 11;16(9):e114040. doi: 10.1136/bmjopen-2025-114040 (PMC13583865; doi:10.1136/bmjopen-2025-114040)
Supplement: Supplementary file 1 [file bmjopen-2025-114040.reviewer_comments.pdf]

PEER REVIEW HISTORY

BMJ Open publishes all reviews undertaken for accepted manuscripts. Reviewers are asked to complete a checklist review form and are provided with free text boxes to elaborate on their assessment. These free text comments are reproduced below.

ARTICLE DETAILS

Title (Provisional)

Tranexamic acid to prevent anastomotic leak after rectal cancer surgery: protocol for a feasibility trial with embedded mechanistic analysis of the microbiome

Authors

Helliwell, Jack A; Chilton, Caroline H; Bestall, Janine; Kirby, Andrew; Quirke, Phillip; Wood, Henry M; Stocken, Deborah D; Jayne, David

---

VERSION 1 - REVIEW

**Reviewer**	**1**
**Name**	**Tada, Naoya**
**Affiliation**	**Jikei University Hospital**
**Date**	**23-Mar-2026**
**COI**	**None**

This study addresses an important clinical issue, and the protocol is generally well written. I have several suggestions that may help to further strengthen the study and the protocol.

Major comments

1. The current feasibility trial seems appropriate as an open-label study. However, if the study moves to a phase III trial, it would be helpful to clarify whether the future trial will also be open-label, or whether some form of blinding is being considered. Because outcomes such as anastomotic leak may be influenced by the assessment process, a brief explanation would strengthen the protocol.

2. The progression criteria in Table 2 include recruitment, adherence, and retention, but safety does not seem to be included in the same clear stop/amend/go framework. The protocol would be strengthened by explaining how safety findings will be considered in deciding whether to move to a future phase III trial, including in Table 2.

3. Rectal administration of tranexamic acid through a catheter is not a standard route of administration, so unexpected adverse events, including local or catheter-related complications, may occur. The protocol would be strengthened by clarifying whether any stopping rules or early termination criteria have been set for the current feasibility study,

especially for safety concerns, and who will make the final decision to suspend or stop the trial.

Minor comment

1. Using tumor distance from the anal verge (<8 cm vs ≥8 cm) as a minimization factor seems reasonable. However, it would be helpful if the authors could briefly explain why 8 cm was chosen as the cut-off.

Reviewer **2**

Name **Zhao, Ze-Liang**

Affiliation **Xin Jiang Medical University, Department of Gastrointestinal Surgery, The Affiliated Cancer Hospital of Xinjiang Medical University**

Date **16-Jun-2026**

COI **None**

This is a well-written and scientifically interesting protocol for a randomised feasibility trial evaluating rectally administered tranexamic acid to prevent anastomotic leak after anterior resection for rectal or sigmoid cancer. The study addresses an important clinical problem and is supported by a biologically plausible microbiome-related mechanism. The incorporation of microbiome and qualitative sub-studies strengthens the translational and feasibility value of the protocol. Overall, the manuscript is suitable for publication after minor revisions and clarifications.

1. Clarification of rectal catheter management is needed. The intervention depends heavily on accurate catheter placement and retention until postoperative day 3. The authors should provide more detail on how catheter position will be confirmed, how dislodgement will be managed, whether reinsertion is permitted, and how catheter-related discomfort or complications will be recorded.

2. Safety monitoring and stopping rules should be more explicit. Although the authors state that safety will be reviewed by the Trial Oversight Committee, it would be helpful to specify what types of adverse events, such as thromboembolic events, seizures, severe rectal irritation, bleeding, catheter-related injury, or unexpected surgical complications, would trigger trial suspension, protocol amendment, or non-progression to a phase III trial.

3. The rationale for the TXA dose and schedule should be expanded. The selected regimen of 1000 mg/50 mL administered intra-operatively and on postoperative days 1–3 is reasonable,

but the manuscript would benefit from a clearer justification based on preclinical evidence, prior topical or rectal pharmacokinetic data, and anticipated local exposure at the anastomotic site.

4. Potential microbiome confounders should be addressed in more detail. The microbiome sub-study is a key strength. However, perioperative antibiotics, bowel preparation, neoadjuvant therapy, dietary intake, diverting stoma, and postoperative complications may substantially influence microbial composition and collagenase activity. The authors should clarify how these variables will be collected and considered in exploratory analyses.

5. The open-label design should be further justified with respect to acceptability outcomes. The open-label design is acceptable for a feasibility trial. However, subjective outcomes from patient and healthcare professional interviews may be influenced by knowledge of allocation. The authors should briefly discuss this limitation and explain how qualitative data collection and analysis will minimise expectation or reporting bias.

6. The progression criteria are useful but require additional operational detail. The stop-amend-go criteria for recruitment, adherence, and retention are clearly presented. However, the manuscript should explain how mixed results across domains will be interpreted. For example, if recruitment meets the “go” criterion but adherence or retention falls into the “amend” range, the decision-making process for proceeding to a definitive trial should be more clearly described.

7. Assessment of anastomotic leak should be standardised despite being exploratory. Although the study is not powered to detect differences in leak rates, anastomotic leak remains a clinically important exploratory outcome. The authors should clarify whether leak assessment will be based on routine clinical evaluation only, radiological confirmation, reoperation findings, or independent review, and whether outcome assessors will be blinded where feasible.

8. The qualitative sub-study would benefit from more methodological detail. The proposed sampling strategy is appropriate, but the authors should specify whether interviews will continue until thematic saturation is reached, who will conduct the interviews, whether coding will be performed independently by more than one researcher, and how discrepancies will be resolved.

9. Statistical analysis of feasibility outcomes should be slightly expanded. The authors state that feasibility outcomes will be described using rates, means, and 95% confidence intervals. It would be useful to clarify whether adherence will be summarised per participant and per scheduled dose, and whether feasibility outcomes will also be stratified descriptively by treatment arm.

10. Please consider citing recent evidence on serum nutritional biomarkers for anastomotic leakage after rectal cancer surgery (PMID: 39575267) and inflammation-based hematologic markers in colorectal cancer (PMID: 41291516) to strengthen the Introduction/Discussion on baseline risk stratification for future trials.

11. Minor wording and consistency issues should be corrected. The manuscript should be checked for minor typographical and wording issues. For example, “complimentary biological and experiential perspectives” should likely be revised to “complementary biological and experiential perspectives.” The terms “rectal tranexamic acid,” “rectally administered tranexamic acid,” and “local tranexamic acid” should also be used consistently throughout the manuscript.

VERSION 1 - AUTHOR RESPONSE

Reviewer: 1
Dr. Naoya Tada, Jikei University Hospital

Comments to the Author:
This study addresses an important clinical issue, and the protocol is generally well written. I have several suggestions that may help to further strengthen the study and the protocol.

Major comments
1. The current feasibility trial seems appropriate as an open-label study. However, if the study moves to a phase III trial, it would be helpful to clarify whether the future trial will also be open-label, or whether some form of blinding is being considered. Because outcomes such as anastomotic leak may be influenced by the assessment process, a brief explanation would strengthen the protocol.

Thank you. The primary purpose of the present study is to evaluate the feasibility, safety and acceptability of rectally administered tranexamic acid, and an open-label design was considered the most appropriate approach for these objectives. The design of any future phase III trial, including whether blinding is feasible and appropriate, will be informed by the findings of this feasibility study and has therefore not been prespecified. We have not amended the manuscript, as discussion of the design of a future definitive trial is beyond the scope of this feasibility protocol.

2. The progression criteria in Table 2 include recruitment, adherence, and retention, but safety does not seem to be included in the same clear stop/amend/go framework. The protocol would be strengthened by explaining how safety findings will be considered in deciding whether to move to a future phase III trial, including in Table 2.

Thank you. We have clarified in the manuscript and the Table 2 legend that safety is intentionally considered outside the quantitative stop-amend-go framework and will be assessed by the independent Trial Oversight Committee when determining progression to a definitive trial.

- Methods. Safety is not included within the stop-amend-go framework because progression cannot be determined using numerical thresholds alone. The implications of adverse events depend on their frequency, severity, expectedness and causality. Accordingly, cumulative safety data, including that related to the medicinal product and rectal catheter, will be reviewed by the independent Trial Oversight Committee throughout the study and at study completion to determine whether any safety concerns preclude progression to a definitive phase III trial.
- Table 2. Safety will be considered separately through independent review of cumulative adverse event data by the Trial Oversight Committee and will inform overall progression decision.

3. Rectal administration of tranexamic acid through a catheter is not a standard route of administration, so unexpected adverse events, including local or catheter-related complications, may occur. The protocol would be strengthened by clarifying whether any stopping rules or early termination criteria have been set for the current feasibility study, especially for safety concerns, and who will make the final decision to suspend or stop the trial.

Thank you. We have clarified the trial governance arrangements by stating that, should significant safety concerns arise, the Trial Oversight Committee may recommend modification, temporary suspension, or early termination to the Sponsor.

- Trial management and oversight. Should significant safety concerns arise, the TOC may recommend modification, temporary suspension, or early termination of the study to the Sponsor.

Minor comment

1. Using tumor distance from the anal verge (<8 cm vs ≥8 cm) as a minimization factor seems reasonable. However, it would be helpful if the authors could briefly explain why 8 cm was chosen as the cut-off.

Thank you. An 8cm cut-off was selected to distinguish low from mid/high rectal tumours. Lower rectal tumours are associated with lower anastomoses and a higher risk of anastomotic leak, making this a clinically relevant minimisation factor.

Reviewer: 2
Dr. Ze-Liang Zhao, Xin Jiang Medical University

Comments to the Author:
This is a well-written and scientifically interesting protocol for a randomised feasibility trial evaluating rectally administered tranexamic acid to prevent anastomotic leak after anterior resection for rectal or sigmoid cancer. The study addresses an important clinical problem and is supported by a biologically plausible microbiome-related mechanism. The incorporation of microbiome and qualitative sub-studies strengthens the translational and feasibility value of the protocol. Overall, the manuscript is suitable for publication after minor revisions and clarifications.

Thank you.

1. Clarification of rectal catheter management is needed. The intervention depends heavily on accurate catheter placement and retention until postoperative day 3. The authors should provide more detail on how catheter position will be confirmed, how dislodgement will be managed, whether reinsertion is permitted, and how catheter-related discomfort or complications will be recorded.

Thank you. The following information is provided regarding placement of the rectal catheter:

- Intervention. At the end of the procedure, a rectal catheter will be inserted with the tip positioned just proximal to the anastomosis by the operating surgeon, allowing direct delivery of the allocated treatment to the anastomotic site.

In addition, the following information is provided regarding management of rectal catheter dislodgement:

- Intervention. If the rectal catheter becomes dislodged before all scheduled doses have been given, treatment will be discontinued, and treatment duration recorded.

Regarding safety monitoring related to the investigational medicinal product (IMP) and the rectal catheter, the following information is included:

- Study outcomes and progression criteria. Safety is not included within the stop-amend-go framework because progression cannot be determined using numerical thresholds alone. The implications of adverse events depend on their frequency, severity, expectedness and causality. Accordingly, cumulative safety data, including that related to medicinal product and rectal catheter, will be reviewed by the

independent Trial Oversight Committee throughout the study and at study completion to determine whether any safety concerns preclude progression to a definitive phase III trial.

2. Safety monitoring and stopping rules should be more explicit. Although the authors state that safety will be reviewed by the Trial Oversight Committee, it would be helpful to specify what types of adverse events, such as thromboembolic events, seizures, severe rectal irritation, bleeding, catheter-related injury, or unexpected surgical complications, would trigger trial suspension, protocol amendment, or non-progression to a phase III trial.

Thank you. We agree that safety oversight is an important aspect of the study. However, we have not specified individual adverse events that would automatically trigger trial suspension, protocol amendment or non-progression, as these decisions cannot be determined by predefined event types alone. Instead, cumulative safety data, including frequency, severity, expectedness and causality of adverse events, will be reviewed by the independent Trial Oversight Committee throughout the study and at study completion. Any recommendation regarding protocol amendment, trial suspension, early termination or progression to a definitive phase III trial will be based on the overall safety profile.

3. The rationale for the TXA dose and schedule should be expanded. The selected regimen of 1000 mg/50 mL administered intra-operatively and on postoperative days 1–3 is reasonable, but the manuscript would benefit from a clearer justification based on preclinical evidence, prior topical or rectal pharmacokinetic data, and anticipated local exposure at the anastomotic site.

Thank you for this helpful comment. The rationale for the selected dose and treatment schedule is described in the manuscript and is based on the available preclinical and clinical evidence. As discussed, there are currently no published rectal pharmacokinetic data to inform dosing at the anastomotic site. The selected regimen was therefore chosen to provide sustained local exposure during the early postoperative period, when anastomotic healing is most vulnerable, while remaining within the established safety profile of tranexamic acid. We have not expanded this section further, as there are limited additional data available to support a more detailed justification.

4. Potential microbiome confounders should be addressed in more detail. The microbiome sub-study is a key strength. However, perioperative antibiotics, bowel preparation, neoadjuvant therapy, dietary intake, diverting stoma, and postoperative complications may substantially influence microbial composition and collagenase activity. The authors should clarify how these variables will be collected and considered in exploratory

analyses.

Thank you. We agree that these factors may influence the gut microbiome. The protocol already includes collection of relevant perioperative variables, including bowel preparation, perioperative antibiotic use, neoadjuvant therapy, diverting stoma formation and postoperative complications. These variables will be considered in exploratory analyses when interpreting microbiome composition and collagenase activity. Given the exploratory nature of the microbiome sub-study and the feasibility design, we have not prespecified adjusted analyses for individual confounding factors.

5. The open-label design should be further justified with respect to acceptability outcomes. The open-label design is acceptable for a feasibility trial. However, subjective outcomes from patient and healthcare professional interviews may be influenced by knowledge of allocation. The authors should briefly discuss this limitation and explain how qualitative data collection and analysis will minimise expectation or reporting bias.

Thank you for this helpful comment. The absence of blinding is not anticipated to impact interpretation of any of the predefined progression criteria, which are based on quantitative feasibility outcomes. We agree that the open-label design may influence subjective responses during the qualitative interviews. However, these interviews are exploratory in nature and are intended to provide contextual insights into the acceptability of the intervention. They do not contribute directly to the predefined progression criteria.

6. The progression criteria are useful but require additional operational detail. The stop-amend-go criteria for recruitment, adherence, and retention are clearly presented. However, the manuscript should explain how mixed results across domains will be interpreted. For example, if recruitment meets the "go" criterion but adherence or retention falls into the "amend" range, the decision-making process for proceeding to a definitive trial should be more clearly described.

Thank you. The following information is now provided:

- Study outcomes and progression criteria. The decision to progress will be based on the overall assessment across all feasibility domains, informed by both quantitative thresholds and safety review. No single feasibility domain will determine progression in isolation. Where one or more domains fall within the 'amend' range, progression

will be informed by consideration of the underlying reasons and whether these can be addressed through modifications to the trial design or conduct.

7. Assessment of anastomotic leak should be standardised despite being exploratory. Although the study is not powered to detect differences in leak rates, anastomotic leak remains a clinically important exploratory outcome. The authors should clarify whether leak assessment will be based on routine clinical evaluation only, radiological confirmation, reoperation findings, or independent review, and whether outcome assessors will be blinded where feasible.

Thank you. Anastomotic leak is an exploratory outcome and will be identified according to the predefined study definition through routine clinical assessment. The diagnosis is confirmed by routine clinical investigations, including radiological findings and/or findings at reoperation where appropriate. As this is an objective clinical outcome, blinded outcome assessment is not considered necessary.

8. The qualitative sub-study would benefit from more methodological detail. The proposed sampling strategy is appropriate, but the authors should specify whether interviews will continue until thematic saturation is reached, who will conduct the interviews, whether coding will be performed independently by more than one researcher, and how discrepancies will be resolved.

Thank you. We have expanded the description of the qualitative methodology to clarify that interviews will be conducted by a study investigator trained in qualitative research methods. The qualitative sub-study has a prespecified sample size and therefore interviews will not continue until thematic saturation is reached. Independent double coding was not prespecified within the study protocol and has therefore not been added to the manuscript.

- Qualitative sub-study. Semi-structured interviews, guided by pre-designed topic guides, will be conducted by a study investigator trained in qualitative research methods in hospital or outpatient settings and will last approximately 30-45 minutes. Interviews will be audio-recorded, transcribed verbatim, and thematically analysed to identify key factors influencing trial feasibility and acceptability [14].

9. Statistical analysis of feasibility outcomes should be slightly expanded. The authors state that feasibility outcomes will be described using rates, means, and 95% confidence intervals. It would be useful to clarify whether adherence will be summarised per

participant and per scheduled dose, and whether feasibility outcomes will also be stratified descriptively by treatment arm.

Thank you. We have expanded the statistical analysis plan section to clarify that feasibility outcomes will be summarised descriptively overall and by treatment allocation, where appropriate. The predefined adherence outcome remains the mean number of scheduled treatment doses received by participant, as described in the progression criteria.

- Statistical analysis plan. All analyses will follow a pre-determined statistical analysis plan and will be overseen by a qualified medical statistician [15]. Baseline characteristics, recruitment, feasibility data, and clinical outcomes will be presented descriptively as rates (categorical) and means (continuous) with 95% confidence intervals. Feasibility outcomes will be summarised overall and descriptively by treatment allocation, where appropriate.

10. Please consider citing recent evidence on serum nutritional biomarkers for anastomotic leakage after rectal cancer surgery (PMID: 39575267) and inflammation-based hematologic markers in colorectal cancer (PMID: 41291516) to strengthen the Introduction/Discussion on baseline risk stratification for future trials.

Thank you for this helpful suggestion. We agree that these studies make an important contribution to the literature on risk prediction and prognostic biomarker development in colorectal surgery. However, the primary aim of the present study is to evaluate the feasibility, safety and acceptability of a microbiome-targeted intervention, rather than to investigate baseline risk stratification or develop prediction models. We have therefore not incorporated these references. Instead, we have included two recent publications that are directly relevant to the biological rationale underpinning this study, namely the role of the gut microbiome in anastomotic leak and the potential for microbiome modulation as a preventative strategy:

- Helliwell JA, Quyn A, Jayne DG. *Anastomotic leak after colorectal surgery: current landscape and future directions.* Br J Surg. 2026;113:znag041. doi:10.1093/bjs/znag041.
- Helliwell JA, Sciberras P, Dosis A, et al. *Modulation of the gut microbiota as a novel strategy to prevent anastomotic leak after colorectal surgery: Systematic scoping review.* Colorectal Dis. 2026;28:e70472. doi:10.1111/codi.70472.

11. Minor wording and consistency issues should be corrected. The manuscript should be checked for minor typographical and wording issues. For example, “complimentary biological and experiential perspectives” should likely be revised to “complementary biological and experiential perspectives.” The terms “rectal tranexamic acid,” “rectally administered tranexamic acid,” and “local tranexamic acid” should also be used

consistently throughout the manuscript.

Thank you for highlighting these points. We have carefully proofread the manuscript and corrected minor typographical and wording inconsistencies throughout, including revising “complimentary” to “complementary.” We have also standardised the terminology used to describe the intervention to improve consistency across the manuscript.

VERSION 2 - REVIEW

Reviewer **1**

Name **Tada, Naoya**

Affiliation **Jikei University Hospital**

Date **27-Jul-2026**

COI

Comments to the Author:

The authors have adequately addressed the points raised in the previous review. I have no further comments.

Reviewer **2**

Name **Zhao, Ze-Liang**

Affiliation **Xin Jiang Medical University, Department of Gastrointestinal Surgery, The Affiliated Cancer Hospital of Xinjiang Medical University**

Date **13-Aug-2026**

COI

Accept.
