# Supplementary material for: Tranexamic acid to prevent anastomotic leak after rectal cancer surgery: protocol for a feasibility trial with embedded mechanistic analysis of the microbiome
Source: BMJ Open. 2026 Sep 11;16(9):e114040. doi: 10.1136/bmjopen-2025-114040 (PMC13583865; doi:10.1136/bmjopen-2025-114040)
Supplement: Supplementary file 2 [file bmjopen-16-9-s001.docx]

**SUPPLEMENTARY FILE**

**Role of the Sponsor and Funders**

The University of Leeds (Email: [leedsth-tr.qamonitors@nhs.net](mailto:leedsth-tr.qamonitors@nhs.net)) is the trial sponsor and holds overall responsibility for ensuring the study is conducted in accordance with regulatory and ethical standards. The Sponsor does not have a role in the day-to-day conduct of the trial, analysis, or interpretation of data, or the reporting of results, which remain under the control of the trial management group. The study funder (NIHR) similarly has no authority over trial design, conduct, data analysis, or the decision to submit results for publication.

**Protocol Version and Amendments**

The current protocol version is 4.0 (dated 25 April 2025). REC and MHRA approval were granted on 30^th^ October 2024, with site-specific approvals enabling recruitment from 7^th^ January 2025. During the first five months of study opening, two substantial amendments were submitted and approved. The first addressed the reporting of expected surgical adverse events, while the second revised the wording of eligibility criteria to include patients with high-grade dysplasia and endoscopic or radiological features suggestive of cancer, ensuring that clinically appropriate patients were not excluded. Additional minor clarifications were also included in these amendments.

**Communication of Protocol Amendments**

All substantial amendments, once approved by REC and MHRA, are communicated to trial personnel at St James’s University Hospital. Staff are provided with updated protocol documents and a summary of the changes. Amendments are also recorded in the trial master file to maintain an accurate record of all protocol versions and modifications.

**Ancillary and Post-Trial Care**

All participants will continue to receive standard NHS clinical care throughout and following their participation in the trial. Any adverse events arising during the study will be managed according to local clinical protocols, with appropriate follow-up.

The University of Leeds, as the trial sponsor, provides insurance to cover liabilities arising from negligent harm. In certain circumstances, insurance may also cover claims arising from non-negligent harm. Clinical negligence indemnification rests with the participating NHS Trust under standard NHS arrangements.

**Supplementary Table 1. Schedule of events.**

|  |  | **Pre-admission** | | | **Admission to hospital** | | | | | | | **Post-admission** | |  |
| --- | --- | --- | --- | --- | --- | --- | --- | --- | --- | --- | --- | --- | --- | --- |
|  |  | **Colorectal MDT** | **Pre-operative visit 1** | **Pre-operative visit 2** | **Day of surgery** | **POD 1** | **POD 2** | **POD 3** | **POD 4** | **POD 5** | **Day of discharge** | **Clinic appointment** | **Month 3** |  |
|  | | **Feasibility study** | | | | | | | | | | | | |
|  | Identification of eligible patients | x |  |  |  |  |  |  |  |  |  |  |  |  |
|  | Initial approach & information given |  | x | x |  |  |  |  |  |  |  |  |  |  |
|  | Written informed consent |  |  | x |  |  |  |  |  |  |  |  |  |  |
|  | Randomisation |  |  | x |  |  |  |  |  |  |  |  |  |  |
|  | Pregnancy test ^1^ |  |  |  | x |  |  |  |  |  |  |  |  |  |
|  | Surgery |  |  |  | x |  |  |  |  |  |  |  |  |  |
|  | Placement of rectal  catheter |  |  |  | x |  |  |  |  |  |  |  |  |  |
|  | TXA or Control treatment |  |  |  | x | x | x | x |  |  |  |  |  |  |
|  | Removal of rectal catheter |  |  |  |  |  |  | x |  |  |  |  |  |  |
|  | AEs/SAEs check ^2^ |  |  |  | x | | | | | | | x |  |  |
|  | | **Microbiome sub-study** | | | | | | | | | | | | |
|  | Collection of rectal microbiome sample |  |  | x | x |  |  | x | | |  |  |  |  |
|  | | **Qualitative sub-study** | | | | | | | | | | | | |
|  | Qualitative interviews |  |  |  |  |  |  |  |  |  | x | | |  |
|  | | **Data collection** | | | | | | | | | | | | |
|  | CRF A: Participant Characteristics^3^ |  |  | x |  |  |  |  |  |  |  |  |  |  |
|  | CRF B: Cancer Characteristics^3^ |  |  | x |  |  |  |  |  |  |  |  |  |  |
|  | CRF C: Surgical Treatment Characteristics |  |  |  | x |  |  |  |  |  |  |  |  |  |
|  | CRF D: Trial Treatment^4^ |  |  |  | x | | | | | | | x |  |  |
|  | CRF E: Clinical follow-up ^5^ |  |  |  |  | x | | | | | | | |  |

Timeline of key study procedures, including pre-admission, admission, and post-admission assessments for the feasibility trial, microbiome sub-study, and qualitative sub-study. Abbreviations: CRF = case report form; MDT = multidisciplinary team; TXA = tranexamic acid; IMP = investigational medicinal product; AE = adverse event; SAE = serious adverse event; POD = post-operative day.

^1^ Pregnancy test should be performed within 24 hours prior to commencement of IMP administration for women of childbearing potential.

^2^ AEs/SAEs should be monitored until discharge, including daily monitoring until 24 hours after IMP administration, as well as during the post-operative clinic appointment.

^3^ Written informed consent must be obtained prior to collecting data for CRF A and B as well as the pre-operative microbiome sample.

^4^ Data for CRF D will be collected daily until discharge from hospital and at the post-operative clinic appointment.

^5^ Clinical follow-up data for CRF E will be collected up to 90 days after surgery for all participants.

**Supplementary Table 2. SPIRIT 2025 checklist of items to address in a randomized trial protocol.**

| **Section / Topic** | **No** | **SPIRIT 2025 checklist item description** | **Reported on page no.** |
| --- | --- | --- | --- |
| **Administrative information** | | |  |
| Title and structured summary | 1a | Title stating the trial design, population, and interventions, with identification as a protocol | Page 1 |
|  | 1b | Structured summary of trial design and methods, including items from the World Health Organization Trial Registration Data Set | Page 2 |
| Protocol version | 2 | Version date and identifier | Supplementary file |
| Roles and responsibilities | 3a | Names, affiliations, and roles of protocol contributors | Page 1 |
|  | 3b | Name and contact information for the trial sponsor | Supplementary file |
|  | 3c | Role of trial sponsor and funders in design, conduct, analysis, and reporting of trial; including any authority over these activities | Supplementary file |
|  | 3d | Composition, roles, and responsibilities of the coordinating site, steering committee, endpoint adjudication committee, data management team, and other individuals or groups overseeing the trial, if applicable | Page 9-10 |
| **Open science** | | |  |
| Trial registration | 4 | Name of trial registry, identifying number (with URL), and date of registration. If not yet registered, name of intended registry | Page 10 |
| Protocol and statistical analysis plan | 5 | Where the trial protocol and statistical analysis plan can be accessed | Page 9 |
| Data sharing | 6 | Where and how the individual de-identified participant data (including data dictionary), statistical code, and any other materials will be accessible | Page 1 |
| Funding and conflicts of interest | 7a | Sources of funding and other support (e.g., supply of drugs) | Page 1 |
|  | 7b | Financial and other conflicts of interest for principal investigators and steering committee members | Page 1 |
| Dissemination policy | 8 | Plans to communicate trial results to participants, healthcare professionals, the public, and other relevant groups (e.g., reporting in trial registry, plain language summary, publication) | Page 10 |
| **Introduction** | | |  |
| Background and rationale | 9a | Scientific background and rationale, including summary of relevant studies (published and unpublished) examining benefits and harms for each intervention | Page 3 |
|  | 9b | Explanation for choice of comparator | Page 7 |
| Objectives | 10 | Specific objectives related to benefits and harms | Page 4 |
| **Methods: Patient and public involvement, trial design** | | |  |
| Patient and public involvement | 11 | Details of, or plans for, patient or public involvement in the design, conduct, and reporting of the trial | Page 10 |
| Trial design | 12 | Description of trial design including type of trial (e.g., parallel group, crossover), allocation ratio, and framework (e.g., superiority, equivalence, non-inferiority, exploratory) | Page 5 |
| **Methods: Participants, interventions, and outcomes** | | |  |
| Trial setting | 13 | Settings (e.g., community, hospital) and locations (e.g., countries, sites) where the trial will be conducted | Page 5 |
| Eligibility criteria | 14a | Eligibility criteria for participants | Page 5 |
|  | 14b | If applicable, eligibility criteria for sites and for individuals who will deliver the interventions (e.g., surgeons, physiotherapists) | N/A |
| Intervention and comparator | 15a | Intervention and comparator with sufficient details to allow replication including how, when, and by whom they will be administered. If relevant, where additional materials describing the intervention and comparator (e.g., intervention manual) can be accessed | Page 6-7 |
|  | 15b | Criteria for discontinuing or modifying allocated intervention/comparator for a trial participant (e.g., drug dose change in response to harms, participant request, or improving/worsening disease) | Page 7 |
|  | 15c | Strategies to improve adherence to intervention/comparator protocols, if applicable, and any procedures for monitoring adherence (e.g., drug tablet return, sessions attended) | Page 7 |
|  | 15d | Concomitant care that is permitted or prohibited during the trial | Page 6-7 |
| Outcomes | 16 | Primary and secondary outcomes, including the specific measurement variable (e.g., systolic blood pressure), analysis metric (e.g., change from baseline, final value, time to event), method of aggregation (e.g., median, proportion), and time point for each outcome | Page 7-8 |
| Harms | 17 | How harms are defined and will be assessed (e.g., systematically, non-systematically) | Page 7 |
| Participant timeline | 18 | Time schedule of enrollment, interventions (including any run-ins and washouts), assessments, and visits for participants. A schematic diagram is highly recommended (see Figure) | Page 5 |
| Sample size | 19 | How sample size was determined, including all assumptions supporting the sample size calculation | Page 8 |
| Recruitment | 20 | Strategies for achieving adequate participant enrollment to reach target sample size | Page 6 |
| **Methods: Assignment of interventions** | | |  |
| Randomization: |  |  |  |
| Sequence generation | 21a | Who will generate the random allocation sequence and the method used | Page 6 |
|  | 21b | Type of randomization (simple or restricted) and details of any factors for stratification. To reduce predictability of a random sequence, other details of any planned restriction (e.g., blocking) should be provided in a separate document that is unavailable to those who enroll participants or assign interventions | Page 6 |
| Allocation concealment  mechanism | 22 | Mechanism used to implement the random allocation sequence (e.g., central computer/telephone; sequentially numbered, opaque, sealed containers), describing any steps to conceal the sequence until interventions are assigned | Page 6 |
| Implementation | 23 | Whether the personnel who will enroll and those who will assign participants to the interventions will have access to the random allocation sequence | Page 6 |
| Blinding | 24a | Who will be blinded after assignment to interventions (e.g., participants, care providers, outcome assessors, data analysts) | Page 6 |
|  | 24b | If blinded, how blinding will be achieved and description of the similarity of interventions | N/A |
|  | 24c | If blinded, circumstances under which unblinding is permissible, and procedure for revealing a participant’s allocated intervention during the trial | N/A |
| **Methods: Data collection, management, and analysis** | | |  |
| Data collection methods | 25a | Plans for assessment and collection of trial data, including any related processes to promote data quality (e.g., duplicate measurements, training of assessors) and a description of trial instruments (e.g., questionnaires, laboratory tests) along with their reliability and validity, if known. Reference to where data collection forms can be accessed, if not in the protocol | Page 9 |
|  | 25b | Plans to promote participant retention and complete follow-up, including list of any outcome data to be collected for participants who discontinue or deviate from intervention protocols | Page 7 |
| Data management | 26 | Plans for data entry, coding, security, and storage, including any related processes to promote data quality (e.g., double data entry; range checks for data values). Reference to where details of data management procedures can be accessed, if not in the protocol | Page 9 |
| Statistical methods | 27a | Statistical methods used to compare groups for primary and secondary outcomes, including harms | Page 9 |
|  | 27b | Definition of who will be included in each analysis (e.g., all randomized participants), and in which group | Page 9 |
|  | 27c | How missing data will be handled in the analysis | Page 9 |
|  | 27d | Methods for any additional analyses (e.g., subgroup and sensitivity analyses) | Page 9 |
| **Methods: Monitoring** | | |  |
| Data monitoring committee | 28a | Composition of data monitoring committee (DMC); summary of its role and reporting structure; statement of whether it is independent from the sponsor and funder; conflicts of interest and reference to where further details about its charter can be found, if not in the protocol. Alternatively, an explanation of why a DMC is not needed | Page 10 |
|  | 28b | Explanation of any interim analyses and stopping guidelines, including who will have access to these interim results and make the final decision to terminate the trial | Page 10 |
| Trial monitoring | 29 | Frequency and procedures for monitoring trial conduct. If there is no monitoring, give explanation | Page 10 |
| **Ethics** | | |  |
| Research ethics approval | 30 | Plans for seeking research ethics committee/institutional review board approval | Page 10 |
| Protocol amendments | 31 | Plans for communicating important protocol modifications to relevant parties | Supplementary file |
| Consent or assent | 32a | Who will obtain informed consent or assent from potential trial participants or authorized proxies, and how | Page 6 |
|  | 32b | Additional consent provisions for collection and use of participant data and biological specimens in ancillary studies, if applicable | Page 6 |
| Confidentiality | 33 | How personal information about potential and enrolled participants will be collected, shared, and maintained in order to protect confidentiality before, during, and after the trial | Page 9 |
| Ancillary and post-trial care | 34 | Provisions, if any, for ancillary and post-trial care, and for compensation to those who suffer harm from trial participation | Supplementary file |
